# A Polyclonal Antibody Raised against the *Burkholderia cenocepacia* OmpA-like Protein BCAL2645 Impairs the Bacterium Adhesion and Invasion of Human Epithelial Cells In Vitro

**DOI:** 10.3390/biomedicines9121788

**Published:** 2021-11-29

**Authors:** António M. M. Seixas, Sílvia A. Sousa, Joana R. Feliciano, Sara C. Gomes, Mirela R. Ferreira, Leonilde M. Moreira, Jorge H. Leitão

**Affiliations:** 1 Department of Bioengineering, IBB—Institute for Bioengineering and Biosciences, Instituto Superior Técnico, Universidade de Lisboa, Av. Rovisco Pais, 1049-001 Lisboa, Portugal; antonio.seixas@tecnico.ulisboa.pt (A.M.M.S.); joana.feliciano@tecnico.ulisboa.pt (J.R.F.); saracgomes@tecnico.ulisboa.pt (S.C.G.); mirela.ferreira@tecnico.ulisboa.pt (M.R.F.); lmoreira@tecnico.ulisboa.pt (L.M.M.); 2 Associate Laboratory, i4HB—Institute for Health and Bioeconomy at Instituto Superior Técnico, Universidade de Lisboa, Av. Rovisco Pais, 1049-001 Lisboa, Portugal

**Keywords:** *Burkholderia cenocepacia*, OmpA-like protein, cystic fibrosis, human epithelial cells, immunotherapies, antibody

## Abstract

Respiratory infections by bacteria of the *Burkholderia cepacia* complex (Bcc) remain a life threat to cystic fibrosis (CF) patients, due to the faster lung function decline and the absence of effective eradication strategies. Immunotherapies are regarded as an attractive alternative to control and reduce the damages caused by these infections. In this work, we report the cloning and functional characterization of the OmpA-like BCAL2645 protein, previously identified and found to be immunoreactive against sera from CF patients with a record of Bcc infections. The BCAL2645 protein is shown to play a role in biofilm formation, adherence to mucins and invasion of human lung epithelial cells. The expression of the BCAL2645 protein was found to be increased in culture medium, mimicking the lungs of CF patients and microaerophilic conditions characteristic of the CF lung. Moreover, a polyclonal antibody raised against BCAL2645 was found to inhibit, by about 75 and 85%, the ability of *B. cenocepacia* K56-2 to bind and invade in vitro CFBE41o- human bronchial epithelial cells. These results highlight the potential of anti-BCAL2645 antibodies for the development of passive immunization therapies to protect CF patients against Bcc infections.

## 1. Introduction

The *Burkholderia cepacia* complex (Bcc) is a group of related species that emerged in the early 1980s as important respiratory pathogens, mostly affecting patients suffering from cystic fibrosis (CF) [1]. This bacterial complex currently encompasses 23 species [2,3,4], all capable of causing infections, the species *B. cenocepacia* and *B. multivorans* being the ones more commonly recovered from CF patients [5]. These bacteria are found in diverse natural environments, but are also capable of surviving in human-made environments, like hospital settings, medical devices and pharmaceutical aqueous solutions, increasing the risk of hospital outbreaks [6]. Bcc strains hardly cause infections in healthy individuals, as the normal mucociliary activity is able to clear the bacteria [7]. However, these bacteria are opportunistic pathogens capable of causing life-threatening respiratory tract infections among immunocompromised patients, patients suffering from chronic granulomatous disease and, particularly, among patients with CF. These infections are characterized by an increased decline of the pulmonary function, associated with chronic infection and exacerbation periods [8]. The development of a fatal acute pneumonia, known as the cepacia syndrome, can also occur [9]. Bcc bacteria are well-known for their intrinsic resistance to a broad spectrum of antimicrobials [10,11], and often in vitro susceptibility does not correspond to in vivo susceptibility. The in vivo fail of antimicrobials is linked to many factors, both from the bacteria and the host [12,13,14]. Several mechanisms have been identified in Bcc as responsible for acquired resistance, like the increased activity of efflux pumps, mutations in targets, enzymatic inactivation or modification of the antimicrobial, and reduced cell permeability [15,16]. The ability to rapidly spread from patient to patient, the extent of virulence factors and the intrinsic resistance to the majority of clinically available antimicrobials makes Bcc chronic infections highly dangerous, nearly untreatable [15] and with an unpredictable outcome [17]. Currently there is no effective strategy to eradicate Bcc infection from CF patients [18]. The most common strategies rely on in vitro susceptibility testing followed by a combination of two antibiotics, although these rarely result in the eradication of the pathogen. This lack of effective therapies and the increasing resistance to antimicrobials in CF pathogens [19] highlights the need for new strategies to eradicate Bcc infections [20]. Immunotherapies are therefore regarded has having the best chances of long-term success in the protection against Bcc infections [20,21]. The discovery of possible new targets for the development of immunoprotective strategies against Bcc prompted us to uncover surface exposed proteins from the highly transmissible epidemic Bcc strain *B. cenocepacia* J2315 [22]. A surfomics approach allowed the isolation and identification of the surface exposed moieties of proteins expressed in *B. cenocepacia* J2315 [23]. Sixteen of these surface exposed proteins were predicted to be immunogenic, and among them the BCAL2645 protein was chosen for further studies. The protein belongs to the OmpA family and was found to be immunoreactive against the sera of CF patients with a record of Bcc infections, demonstrating that the protein elicits IgG production [23]. The protein was chosen due to the relevance of OmpA proteins, a family of outer membrane, surface exposed and heat-modifiable porins that are abundant in most of Gram-negative bacteria. These proteins are anchored in the outer membrane forming β-barrel structures composed of 8 to 26 strands, with extracellular large loops and periplasmatic short loops. These structural characteristics confer to OmpA proteins a high stability and ability to withstand harsh environments [24]. Despite the different sequences and functions of OmpAs, they share similar structures and biological functions in pathogenesis, such as bacterial adhesion and invasion of the host, intracellular survival, biofilm formation, stimulation of proinflammatory cytokines and evasion of host defenses [25,26,27,28,29,30]. OmpA proteins also play important roles in the bacterial cell physiology, including active and passive ion and solute transport, signal transduction, catalysis, membrane structure and stability [31]. In addition, their surface exposure renders these proteins targets of the host immune system, capable of activating both innate and adaptive immune mechanisms.

OmpA proteins have been studied as potential vaccine candidates to prevent infections by several pathogens like *Pseudomonas aeruginosa*, *Salmonella* spp., *Mannheimia haemolytica* and others [28]. OmpA proteins were also successfully used to develop vaccines to prevent Lyme disease [32]. The choice of surface-exposed proteins as targets for the development of immunological strategies to combat infections is owed to the fact that these proteins play essential roles within the bacterial cell biology, ranging from signal transduction to translocation of solutes and proteins [33]. Moreover, surface-exposed proteins are at the forefront of the initial contacts with the host, and play critical roles in the pathogenesis process, such as the adhesion to structures and cells of the host, important for the colonization and possible invasion of the host tissues and cells. The delivery of virulence factors and toxins to the host cell and resistance to the host immune system strongly facilitates the colonization and subsequent internalization into the host cells [34]. In the case of Bcc, the adhesion and invasion of host epithelial cells is considered one of the most important steps contributing to their overall virulence. The more virulent Bcc strains obtained from clinical samples have been shown to exhibit extensively higher adhesion and invasion capabilities when compared to environmental strains [35]. In the present work, we have cloned and functionally characterized the OmpA protein BCAL2645. The purified protein was also used to raise polyclonal antibodies, which are here shown to strongly inhibit the ability of *B. cenocepacia* K56-2 to adhere and invade the CFBE41o- human bronchial epithelial cell line.

## 2. Materials and Methods

### 2.1. Bacterial Strains, Plasmids, and Culture Conditions

The bacterial strains and plasmids used in this work are listed in Table 1. When in use, Bcc strains were maintained in PIA (*Pseudomonas* Isolation Agar, BD) plates*. Escherichia coli* strains were maintained in Lennox broth (containing 10 g/L tryptone, 5 g/L yeast extract and 5 g/L NaCl) agar plates. Unless otherwise indicated, liquid cultures were carried out at 37 °C in LB liquid medium supplemented with the appropriate antibiotics for *E. coli* (ampicillin 150 mg/L, chloramphenicol 100 mg/L, kanamycin 50 mg/L, tetracycline 25 mg/L, trimethoprim 100 mg/L), or *Burkholderia* (chloramphenicol 300 mg/L, tetracycline 150 mg/L, trimethoprim 300 mg/L), with orbital agitation (250 rev·min^−1^). Bacterial growth was followed by measuring the cultures optical density at 640 nm (OD_640_). The Artificial Sputum medium (ASM) was used to mimic nutritional conditions encountered by the bacterium in the CF lung and contained 5.0 g/L porcine stomach mucin (Sigma-Aldrich, St. Louis, MO, USA), 4.0 g/L low molecular-weight salmon sperm DNA (Sigma-Aldrich, St. Louis, MO, USA), 5.9 mg/L diethylene triamine pentaacetic acid (DTPA) (Sigma-Aldrich, St. Louis, MO, USA) as iron-chelating agent, 5.0 g/L NaCl (Sigma-Aldrich, St. Louis, MO, USA), 2.2 g/L KCl (Sigma-Aldrich, St. Louis, MO, USA), 5.0 mL/L egg yolk emulsion (phosphatidylcholine as source of lecithin) (Sigma-Aldrich, St. Louis, MO, USA), 5.0 g/L casamino acids (Difco, Detroit, MI, USA)), 1.81 g/L Tris Base (Sigma-Aldrich, St. Louis, MO, USA), and 20 g/L agar (Iberagar, Coina, Portugal) (pH 7.0).

### 2.2. Cell Line and Cell Culture

The CFBE41o- human bronchial epithelial cell line was used in this work. These cells are homozygous for the Δ*F508* mutation of the cyclic AMP activated chloride channel, known as the CF transmembrane conductance regulator (CFTR), causing a decreased transport of chloride ions and defective water transport across the cell, as observed in CF patients. The cell line was previously immortalized and characterized [41]. The cell line was maintained in fibronectin-collagen I-coated flasks with minimum essential medium with Earle’s salt (MEM) (Gibco^®^, Grand Island, NY, USA), supplemented with 10% fetal bovine serum (FBS) (Lonza), 0.292 g/L L-glutamine (Gibco^®^, Grand Island, NY, USA), and penicillin-streptomycin (100 U/mL; Gibco^®^, Grand Island, NY, USA), under humidified atmosphere at 37 °C with 5% CO_2_.

### 2.3. Molecular Biology Techniques

Total DNA was extracted from cells harvested from exponentially growing liquid cultures of *B. cenocepacia* strain K56-2 using the High Pure PCR Template Preparation Kit (Roche, Basel, Switzerland). Plasmid isolation and purification (NZYTech, Lisbon, Portugal), DNA amplification (Thermo Fisher Scientific, San Jose, CA, USA), restriction and T4 DNA ligation (Thermo Fisher Scientific, San Jose, CA, USA), agarose gel electrophoresis, SDS-PAGE and *E. coli* transformation were carried out using standard procedures [42]. The primers used for amplification of *BCAL2645* upstream and downstream regions, and of the *BCAL2645* coding sequence for the construction of a complementation plasmid, are presented in Table 2. Primers were designed based on the genome sequence of *B. cenocepacia* K56-2 (available at [43]).

### 2.4. Construction of a BCAL2645 Deletion Mutant

The chromosomal deletion of the *BURCENK562V_RS26520* gene from the genome of *B. cenocepacia* K56-2 was obtained using the I-SceI homing endonuclease system based on the strategy of Flannagan et al. [44]. This gene is 100% identical to the *BCAL2645* ortholog from *B. cenocepacia* J2315, but due to a difficult genetic handling in the J2315 strain, the mutant was constructed on the K56-2 strain. From this point the gene *BURCENK562V_RS26520* will be referred as *BCAL2645.* The I-SceI homing endonuclease system consists of a suicide vector containing the I-SceI restriction site and a gene encoding the Discosoma red fluorescent protein (*DsRed*), in combination with an unstable plasmid that encodes the I-SceI endonuclease and a modified *sacB* gene enabling the detection of colonies carrying the desired gene deletion. The pAMS6 plasmid, containing the up and downstream regions of the *BCAL2645* gene, was inserted into *B. cenocepacia* K56-2 by triparental mating. The selection of cointegrates was performed using LB plates supplemented with chloramphenicol (300 mg/L). To confirm the integration of the plasmid in the genome, fluorescent microscopy was used to detect the expression of DsRed. The plasmid integration in the genome was also confirmed by PCR, using the primer pair UP-Upstream BCAL2645 and LW- Downstream BCAL2645. The pDAISce-I-SacB plasmid was then mobilized through triparental mating into the previously selected colonies, and the exconjugants were selected in LB plates containing 150 mg/L tetracycline. To confirm the gene deletion, PCR was performed using the same set of primers as before that annealed outside of the deleted region. Removal of the pDAISce-I-SacB plasmid from the mutant was accomplished by growing the strain on the surface of agarized LB without NaCl and supplemented with 5% (*w*/*v*) sucrose, followed by screening of tetracycline sensitive colonies, indicative of pDAI-SceI-SacB loss.

### 2.5. Production of a Goat Polyclonal Antibody Anti-BCAL2645

In order to produce goat polyclonal antibodies against BCAL2645, the protein was produced as a recombinant 6×his-tagged derivative and purified using the methods described by Sousa et al. 2020 [23]. The removal of endotoxin contamination from the protein purified samples was performed using the Detoxi-GelTM endotoxin removing gel (Thermo Scientific) following the manufacturer’s instructions and phosphate buffer 1× (20 mM sodium phosphate, with 500 mM NaCl, pH 7.4) as elution buffer. The production and purification of the polyclonal goat antibody against the purified His_6_-tagged BCAL2645 protein was performed by a commercial company (SICGEN, Portugal). The specificity of the antibody was assessed by Western blot analyses against whole cell extracts of *B. cenocepacia* K56-2.

### 2.6. Western Blot Analyses

Total cell extracts were prepared by harvesting the cells corresponding to a volume of 1 mL aliquot of a culture with an OD_640_ of 0.6. The cells were resuspended in 40 μL of sample buffer (100 mM Tris base pH 6.8, 4 % (*w*/*v*) SDS, 20 % (*v*/*v*) glycerol, 0.2 % (*w*/*v*) bromophenol blue, 200 mM DTT), followed by heating for 5 min at 95 °C. The total cell extracts were then fractionated using 15 % SDS-PAGE. Using a Trans-Blot^®^ SD (BIORAD, Hercules, CA, USA) device, the proteins were transferred to NC membranes (PALL corporation). The membranes were then blocked overnight at 4 °C with 5 % (*w*/*v*) skimmed milk (DIFCO Laboratories, Detroit, MI, USA) in PBS 1×. The membrane was probed with the primary Goat antibody anti-BCAL2645 (1:4000 dilution) for 2 h at room temperature. The HRP-conjugated Mouse anti-Goat IgG antibody (1:10,000 dilution, SANTA CRUZ biotechnology, Dallas, TX, USA) was used as the secondary antibody and was incubated for 1 h at room temperature. The membranes were then treated with the peroxidase substrate ECL (Sigma, St. Louis, MO, USA) and the chemiluminescence signals were detected using a FUSION Solo device (Vilber Lourmat, Collégien, France).

### 2.7. Quantitative Western Blot

The quantification of the BCAL2645 protein expression by *B. cenocepacia* K56-2 was assessed as described before with some modifications [45]. In brief, the bacteria were inoculated in either LB or ASM medium, at an initial OD_640_ of 0.05. Volumes of bacterial cultures grown for 4 h, 6 h, 9 h and 24 h and corresponding to an OD_640_ of 1.8 per mL were centrifuged and the cell pellets were resuspended in 120 μL of sample buffer and heated for 5 min at 95 °C. The protein fractions were separated by SDS-PAGE and transferred onto a nitrocellulose membrane. The membrane was stained with Reversible Protein Stain Kit (Pierce^TM^, Waltham, Massachusetts, USA) for total protein assessment according to the manufacturer’s instructions. The anti-BCAL2645 antibody was then used to detect the BCAL2645 protein by Western Blot. Densitometric analysis was performed using ImageJ software [46], with the background subtracted using a rolling ball radius of 100. The antibody signal was quantified using equal sized boxes drawn around the band. For total protein stains, a thin strip through the center of each lane, from top to bottom, was used. The signal intensity of the target protein in each lane was normalized to the total lane intensity. The relative protein expression levels for each time point were calculated as the ratio between the normalized intensities at a given time point and the control point. The sample collected at the 6 h was defined as the reference. For the microaerophilic conditions, bacterial growth was carried out in 150 μL in the wells of polystyrene microtiter plates with an initial OD_640_ of 0.1 and constant agitation (60 rev.min^−1^). Microaerophilic conditions were obtained using the CampyGenTM Compact Sachet in an OxoidTM Compact Plastic Pouch (Oxoid, Basingstoke, UK). A plate of Muller Hinton agar inoculated with *Campylobacter jejuni* ATCC 33,560 was used as a control of the microaerophilic conditions. Cultures were considered as being at the exponential phase of growth when the OD_640_ reached 1, and at the stationary phase after 24 h of cultivation. These values were chosen after the determination of the growth curves. The sample from the exponential phase for each medium in aerophilic conditions was defined as the reference.

### 2.8. Biofilm Formation Assays

Biofilm formation on the surface of polystyrene was quantified using the dye crystal violet, based on previously described methods [47]. Briefly, polystyrene microtiter plates (Greiner Bio-One) containing LB liquid medium were inoculated with exponential phase bacterial cultures using an initial OD_640_ of 0.05 and incubated for 24 and 48 h at 37 °C without agitation. Quantification of the biofilm formed was accomplished by removing the unattached bacteria after the indicated time of incubation and rinsing the wells three times with deionized water. The attached bacteria were then stained by adding to each well 200 μL of a 1% (*w*/*v*) crystal violet solution and incubating for 15 min at room temperature. After incubation the solution was removed and three gentle washes with deionized water were performed. Biofilm quantification was performed after dissolving the bound crystal violet with 200 μL of 95% ethanol and measuring the absorbance at 595 nm in a microplate reader SpectroStar Nano (BMG Labtech, Ortenberg, Germany). Results are mean values of at least 12 replicates from three independent experiments.

### 2.9. Mucin’s Adhesion Assays

The ability of the WT and the mutant strain with the *BCAL2645* gene deleted to adhere to mucins from porcine stomach (type II) was assessed based on previously described methods, with some modifications [48] as follows: to 96-wells ELISA plates (Greiner Microlon 600, Greiner Bio-One, Kremsmünster, Austria), 200 μL of a solution of Porcine gastric mucin (Sigma-Aldrich) at a concentration of 10 mg/mL in sterile PBS 1× (pH 7.4) was added, followed by overnight incubation at 4 °C. BSA (Bovine Serum Albumin) at 3% (*w*/*v*) was used as control. The wells were then washed twice with sterile PBS 1× and incubated for 1 h at room temperature with 200 μL BSA 3% (*w*/*v*). *Burkholderia cenocepacia* K56-2 and the Δ*BCAL2645* mutant strain were inoculated at an OD_640_ of 0.1. When the cultures reached an OD_640_ of 0.5, the cells were harvested by centrifugation, washed, and suspended in PBS 1× to a final OD_640_ of 0.05 (approximately 5 × 10^7^ cells/mL). Then, 200 μL of each bacterial suspension was added to coated wells and incubated for 1 h at 37 °C. After incubation, the wells were washed twice with PBS 1× to remove unbound bacteria, and 200 μL of 0.5% Triton X-100 was added and agitated for 2 h at room temperature to isolate attached bacteria. The bound bacteria were enumerated by plating serial dilutions onto the surface of LB agar plates. To determine the initial number of bacteria, serial dilutions of the bacterial suspensions were plated onto the surface of LB agar plates. Results are expressed as a ratio of the *B. cenocepacia* K56-2 adhesion to mucins, corrected with the initial bacterial dose applied. When testing the effect of the antibody anti-BCAL2645, the cells were incubated for 1 h at room temperature with an antibody concentration of 0.125 mg/mL prior their addition to the Porcine gastric mucin coated wells.

### 2.10. Adhesion and Invasion of Epithelial Cells

Bacterial adhesion and invasion experiments were carried out using CFBE41o- cystic fibrosis bronchial epithelial cells, based on previously described methods [49,50], with slight modifications. Cells were seeded in 24-well plates (Orange Scientific, Braine-l’Alleud, Belgium) at a concentration of 4 × 10^5^ cells per well for 24 h in supplemented MEM medium (Gibco^®^). The bacterial strains were grown in LB liquid medium at 37 °C with orbital agitation (250 rev·min^−1^) until an OD_640_ of 0.5 was reached and used to infect the 24 h cultivated cells at a multiplicity of infection (MOI) of 50:1. The 24-well plates were then centrifuged at 700× *g* for 5 min. For the adhesion experiments, the cell monolayers were incubated at 37 °C for 30 min in an atmosphere containing 5% CO_2_ after the infection. The wells were then washed 3× with PBS 1×, followed by the addition of 1 mL of lysis buffer (containing 10 mM EDTA and 0.25% (*v*/*v*) Triton X-100) and incubation for further 20 min at 4 °C to lysate the cells. The quantification of the adherent bacteria was performed by plating serial dilutions onto LB agar plates. For the invasion assays, the cells monolayers were incubated with the bacteria for 2 h, allowing the bacterial entry into the cells. The wells were then washed trice with PBS 1× and incubated for an additional 2 h with MEM containing 1 mg/mL amikacin and 1 mg/mL ceftazidime. After this period the supernatants were plated to confirm the effectiveness of the antibiotic treatment. The wells were then washed 3× with PBS and further incubated with 1 mL of lysis buffer for 30 min at 4 °C. After this step plating of serial dilutions was performed to quantify intracellular bacteria. Results were expressed as a ratio of the WT *B. cenocepacia* K56-2 ability to adhere and invade, corrected with the initial bacterial dose applied.

### 2.11. Effect of the Polyclonal Antibody Anti-BCAL2645 on Bacterial Adhesion and Invasion of Epithelial Cells

To test the ability of the antibody anti-BCAL2645 to inhibit the adhesion and invasion of CFBE41o- cystic fibrosis bronchial epithelial cells by the *B. cenocepacia* K56-2 and the Δ*BCAL2645* mutant strain, the bacterial suspensions were incubated for 1 h at room temperature with the anti-BCAL2645 polyclonal goat antibody at a concentration of 0.125 mg/mL prior invasion and adherence experiments. The antibody treated bacterial samples were then washed with PBS and used to infect the cell monolayers as mentioned. Adhesion and invasion were quantified as described above.

### 2.12. Bioinformatics Analyses

Nucleotide and predicted amino acid sequences were analyzed using bioinformatics tools resident at the National Center for Biotechnology Information (NCBI) and the ExPASy-Prosite websites [51]. Searches for homolog sequences within the genomes of *B. cenocepacia* J2315 and other *Burkholderia* strains were carried out using the Burkholderia Genome Database [52].

### 2.13. Statistical Analysis

Results are expressed as mean values of a minimum of three independent experiments ± standard deviations (SD). Statistical analysis was performed using GraphPad Prism software 6.0. Two-way and one-way analysis of variance (ANOVA) were performed to determine statistically significant differences. Results with a *p* value < 0.05 were considered as statistically significant.

## 3. Results

### 3.1. Construction of a BCAL2645 Gene Deletion Mutant from B. cenocepacia K56-2

**Figure 1:**
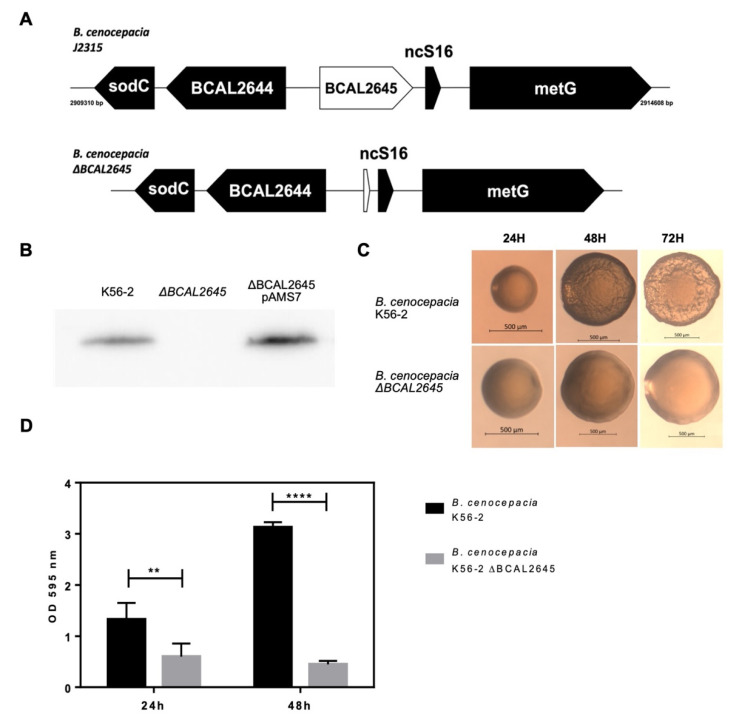
Schematic representation of the region surrounding the *BCAL2645* gene, confirmation of the *BCAL2645* gene deletion, phenotypic alteration in the bacterial colonies and effects of *BCAL2645* gene deletion on *B. cenocepacia* K56-2 ability to form biofilms (**A**) Representation of the genetic organization of the *BCAL2645* gene in the genome of the WT strain and Δ*BCAL2645* deletion strain. (**B**) Western Blot using the anti-BCAL2645 polyclonal antibody against the total protein extracts from *B. cenocepacia* K56-2, the Δ*BCAL2645* mutant strain and the Δ*BCAL2645* strain complemented with the *BCAL2645* gene harbored in plasmid pAMS7. (**C**) Colony morphology change observed in the WT strain where after 48 h a wrinkled colony morphology is observed, while in the Δ*BCAL2645* strain a smooth texture its maintained. Scale bar: 500 μm. (**D**) Evaluation of biofilm formation by the WT strain and the Δ*BCAL2645* strain in the wells of polystyrene microtiter plates, after 24 or 48 h of growth in LB medium at 37 °C without shaking. Error bars represent the standard deviations of the mean values for three independent experiments. (****, *p* < 0.0001; **, *p* < 0.01).

The BCAL2645 protein was identified in a previous work, after shaving the outer membrane proteins from living *B. cenocepacia* J2315 and protein identification by LC-MS/MS [23]. The protein was also demonstrated to elicit IgG antibodies in the sera of CF patients infected with Bcc, highlighting its potential in use for the devolvement of new therapeutic strategies against these bacteria [23]. In order to gain insights into the physiological roles played by this protein, a mutant strain with this gene deleted was constructed, following the strategy of Flannagan et al. [44]. Despite several attempts, the *B. cenocepacia* J2315 mutant preparation was not successful. Therefore, since the gene encoding the *BCAL2645* ortholog from the K56-2 strain is 100% identical to that of the J2315 strain, we have constructed the deletion mutant in the K56-2 strain. This was achieved by first inserting by triparental mating the pAMS6 plasmid containing both the upstream and downstream regions of the gene into *B. cenocepacia* K56-2. The first recombination was confirmed by the red fluorescence exhibited and by PCR. The second recombination was confirmed by PCR with the primers UP and LW-Nested BCAL2645 and western blot using the anti-BCAL2645 goat polyclonal antibody against the total protein extracts of the WT and mutant strain (Figure 1 A,B).

### 3.2. The Deletion of BCAL2645 in B. cenocepacia K56-2 Impairs Biofilm Formation and Alters Colony Morphology

The *B. cenocepacia* K56-2 Δ*BCAL2645* strain was tested for different phenotypes, including the ability to grow in LB and minimal M9 medium and exposure to stress conditions like presence of 0.03% (*w*/*v*) SDS, 426 mM NaCl, 3% (*v*/*v*) ethanol and heat shock of 50 °C. However, no significant differences were observed when comparing results from the mutant to those of the WT strain (data not shown). Resistance to antibiotics like chloramphenicol, trimethoprim and tetracycline have been previously showed to be altered in mutants lacking OmpA-like proteins [53]. However, no differences were observed in the minimal inhibitory concentrations of those antibiotics towards the *B. cenocepacia* K56-2 and the Δ*BCAL2645* deletion mutant. After 48 h of growth on the surface of LB solid medium the WT colonies started to present a wrinkled colony morphology while the Δ*BCAL2645* strain colonies maintained a smooth morphology (Figure 1C).

The effects of the *BCAL2645* gene deletion on the ability of *B. cenocepacia* K56-2 to form biofilms in vitro was also investigated since OmpA-like proteins have been previously demonstrated to be important in adhesion to a variety of surfaces [28]. The results presented in Figure 1D show a significant difference between the WT and the mutant abilities to form biofilms. This difference was much more pronounced at 48 h of incubation, as the WT strain continued to produce more biofilm, while the biofilm produced by the mutant strain slightly decreased. These results suggest that the BCAL2645 protein plays a role in the formation of biofilms, more specifically in later stages, as in its absence bacteria are only able to form a thin biofilm.

### 3.3. The BCAL2645 Protein Is More Expressed under Conditions Mimicking the CF Lung

**Figure 2:**
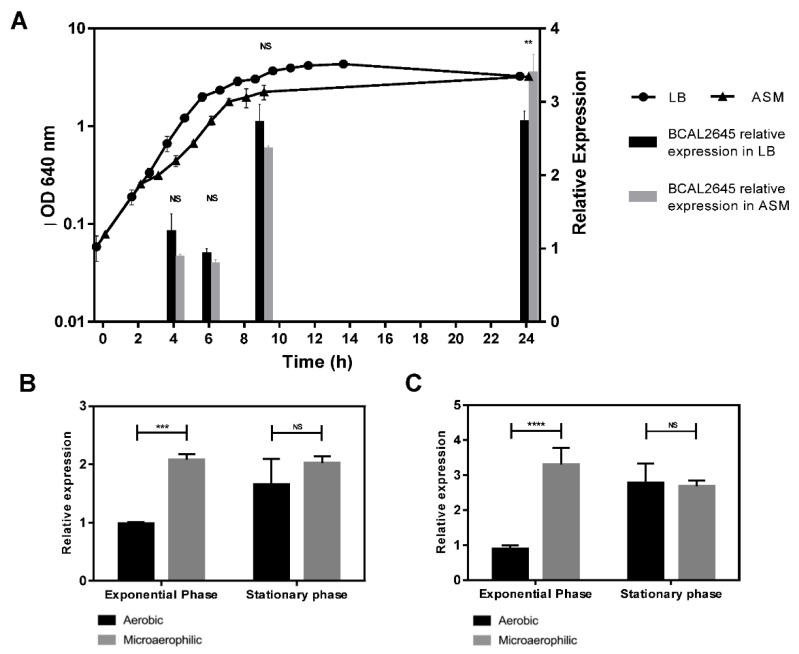
Expression patterns of the BCAL2645 protein in *B. cenocepacia* K56-2. (**A**) Growth curve of *B. cenocepacia* K56-2 in LB and ASM medium with BCAL2645 protein expression levels at the early exponential, exponential, early stationary and late stationary phases, corresponding respectively, to 4, 6, 9 and 24 h of cultivation. The intensity of total proteins and of the bands probed with the anti-BCAL2645 antibody were detected and quantified. Data normalization was performed relative to total proteins for each sample. Samples harvested at 6 h were used as the internal standard reference to determine the relative protein expression levels. Relative amount of BCAL2645 protein in *B. cenocepacia* cultures grown in LB (**B**) or ASM (**C**) medium when grown under microaerophilic or aerobic conditions. Growth experiments were performed in the wells of polystyrene microtiter plates with constant agitation. Microaerophilic conditions were obtained using the CampyGenTM Compact Sachet in OxoidTM Compact Plastic Pouch. Exponential phase was considered when an OD_640_ of 1 was obtained. The stationary phase was reached after 24 h of growth. The value of exponential phase in aerobic conditions was used as the internal standard reference to determine the relative protein levels. Results are representative of three independent experiments. Error bars indicate standard deviation. Statistical analysis was performed by comparison with the 4 h (****, *p* < 0.00001, ***, *p* < 0.001, NS, non-significant).

In a previous work, the RNA transcripts corresponding to the *BCAL2645* gene were found to be more abundant in *B. cenocepacia* J2315 cells at the stationary phase of growth compared with those at the exponential phase of growth. In addition, the *BCAL2645* transcripts were also found to be more abundant when *B. cenocepacia* J2315 was cultivated under microaerophilic conditions [54]. Microaerophilic conditions are recognized as an environmental condition found by bacteria when infecting the CF lungs, as a result of the thickening of the mucus layer on the epithelial cells as the result of the CFTR protein malfunctioning [55]. To understand if the observed variations in the expression of *BCAL2645* at the transcript level are reflected at the protein level, the BCAL2645 protein levels were quantified in cells cultivated in LB and ASM medium at 37 °C and harvested at different phases of growth. The levels of the BCAL2645 protein were quantified along the growth curve of *B. cenocepacia* K56-2 in LB and ASM medium, using equivalent amounts of cells for each time point. The relative amount of BCAL2645 protein was determined by quantitative western blot using the anti-BCAL2645 antibody and the total lane density from the transferred proteins as the internal control for data normalization. The results presented in Figure 2A show that in LB medium the relative expression of the BCAL2645 protein is maximal in cells at the early stationary phase of the growth curve and remained maximal during the stationary phase. In ASM medium, the protein was also found to be more expressed in the stationary phase, reaching higher amounts in ASM medium than in LB at 24 h of growth. The protein was also found in higher amounts in cells grown under microaerophilic conditions compared to aerobic conditions in LB (Figure 2B) and ASM medium (Figure 2C).

### 3.4. The BCAL2645 Protein Affects B. cenocepacia K56-2 Adhesion to Mucins

**Figure 3:**
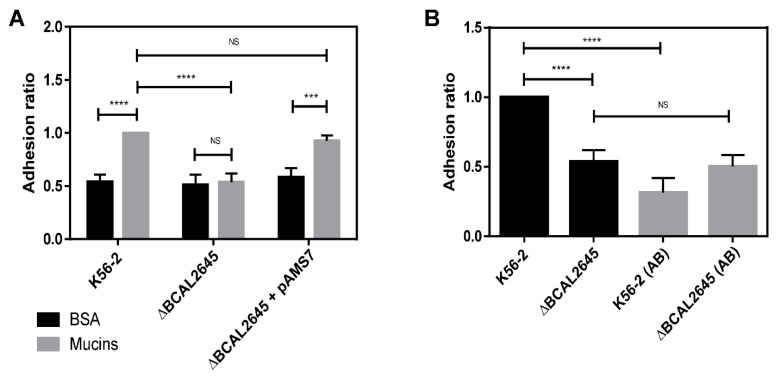
*B. cenocepacia* K56-2 and Δ*BCAL2645* strain adherence to mucins and effect of the antibody anti-BCAL2645 on mucin adhesion in vitro. (**A**) Adherence of *B. cenocepacia* K56-2, Δ*BCAL2645* strain and the mutant strain complemented with the *BCAL2645* gene to BSA (negative control) and mucins. The assays comprised the coating of 96-wells ELISA plates with a solution of mucins or BSA (negative control) with a bacterial incubation period of 1 h. (**B**) Adherence to mucins by the WT strain and the mutant strain after incubation for 1 h with the anti-BCAL2645 antibody (AB) at a concentration of 0.125 mg/mL. Results are expressed as the percentage expressed as a ratio of *B. cenocepacia* K56-2 adherence to mucins. All the results are from at least three independent experiments, and bars indicate SD. (****, *p* < 0.0001; ***, *p* < 0.001, NS, non-significant).

Mucins comprise the major protein component of airway mucus that covers the luminal surface of the respiratory tract, where they can be found as secreted and cell-associated glycoproteins [56,57]. *B. cenocepacia* has been shown to adhere to mucins in CF patients [58] and to have mucin-sulphatase activity that allows the degradation of mucins [59]. The role of BCAL2645 on the adhesion of *B. cenocepacia* K56-2 to mucins was assessed by in vitro assays, comprising the coating of 96-well ELISA plates with a solution of Porcine gastric mucin, followed by quantification of the of bacterial cells that adhered to mucins. Bovine serum albumin fraction V (BSA) was used as a negative control. The results in Figure 3 show a significant decrease in the ability of the Δ*BCAL2645* mutant strain to adhere to mucins when compared to the WT strain. The complementation with the *BCAL2645* gene in plasmid pAMS7 restored the WT phenotype. These results indicate that the protein BCAL2645 plays a role in the adhesion of *B. cenocepacia* K56-2 to mucins. The effect of the polyclonal goat antibody anti-BCAL2645 on the adhesion to mucins was also tested by incubating the cells with the antibody at a concentration of 0.125 mg/mL for one hour prior to the adhesion experiments. This antibody concentration was chosen since further increase beyond this concentration did not significantly change its adhesion inhibitory effect (data not shown). The antibody anti-BCAL2645 reduced by about 70% the number of WT bacteria that adhered to mucins. As expected, the pre-incubation of cells of the mutant strain with the antibody had no effect on adhesion to mucins, indicating that the antibody is specific to the BCAL2645 protein.

### 3.5. The BCAL2645 Protein Contributes to Invasion of Human Bronchial Cell Line CFBE41o-

**Figure 4:**
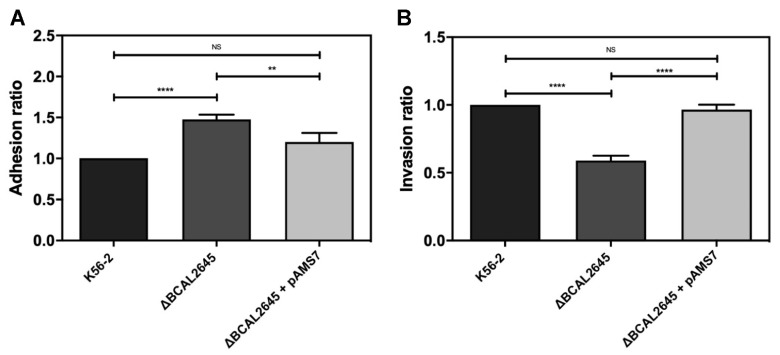
The *BCAL2645* gene is required for invasion of *B. cenocepacia* to the human bronchial epithelial cell line CFBE41o-. (**A**) Adherence to CFBE41o- CF epithelial cell line by the *B. cenocepacia* K56-2, the Δ*BCAL2645* strain and the mutant strain complemented with the *BCAL2645* gene (plasmid pAMS7), expressed as a ratio of *B. cenocepacia* K56-2 adherence. (**B**) Invasion of CFBE41o- CF epithelial cell line by the *B. cenocepacia* K56-2, the Δ*BCAL2645* strain and the mutant strain complemented with the *BCAL2645* gene (plasmid pAMS7), expressed as a ratio of *B. cenocepacia* K56-2 invasion. All the results are from three independent experiments, and bars indicate SD. (****, *p* < 0.0001; **, *p* < 0.01, NS, non-significant).

Since *Burkholderia cenocepacia* is known for its ability to adhere to human cells and survive as an intracellular pathogen [60], we investigated the possible role played by the BCAL2645 protein on adhesion and invasion of cells of the human bronchial epithelial cell line CFBE41o-. This cell line was derived from a CF patient homozygous for the Δ*F508* CFTR mutation [41]. The bacterial strains were used at a multiplicity of infection (MOI) of 50:1. The results presented in Figure 4A show that the Δ*BCAL2645* strain exhibits an increased ability to adhere to the CFBE41o- cells. The complementation of the mutant strain with the *BCAL2645* gene in the pAMS7 plasmid partially restored the WT phenotype. To investigate a possible role of the *BCAL2645* gene on the bacterial ability to invade epithelial cells, CFBE41o- cell monolayers were infected with the WT and the Δ*BCAL2645* strain, and the invasiveness was assessed using a gentamicin invasion assay [50]. The intracellular bacteria were quantified after 4 h of infection. Almost a 50% reduction of the Δ*BCAL2645* mutant ability to invade the epithelial cells was observed when compared to the WT strain (Figure 4B). The insertion of the plasmid pAMS7 (harboring the *BCAL2645* gene) in the mutant strain complemented the phenotype.

### 3.6. A Polyclonal Antibody against BCAL2645 Impairs B. cenocepacia K56-2 Adhesion and Invasion of Epithelial Cells

**Figure 5:**
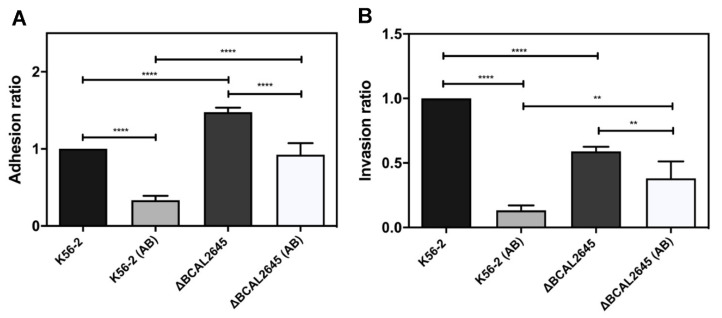
The polyclonal antibody anti-BCAL2645 strongly reduces the ability of *B. cenocepacia* to adhere and invade human bronchial epithelial cell line CFBE41o-. (**A**) Effect of the anti-BCAL2645 goat polyclonal antibody on the ability of *B. cenocepacia* K56-2 and Δ*BCAL2645* strain to adhere to CFBE41o- CF epithelial cell line, expressed as a ratio of *B. cenocepacia* K56-2 adherence. (**B**) Effect of the anti-BCAL2645 goat polyclonal antibody on the ability of *B. cenocepacia* K56-2 and Δ*BCAL2645* strain to invade CFBE41o- CF epithelial cell line. Both strains were incubated with the antibody for 1 h prior to adhesion and invasion experiments. All the results are from three independent experiments, and bars indicate SD. (****, *p* < 0.0001; **, *p* < 0.01, NS, non-significant).

The effect of a polyclonal antibody raised against BCAL2645 in bacterial adhesion and invasion of CFBE41o- cells was investigated. For this purpose, the bacteria were preincubated for 1 h at room temperature with the antibody at a concentration of 0.125 mg/mL prior the contact with the CFBE41o- cells. The pre-incubation with the antibody led to a strong reduction in the ability of the WT strain to adhere to the epithelial cells (close to 75%), and to about 37% reduction in the case of the mutant strain (Figure 5A). The role of the antibody on the invasion of epithelial cells was also investigated by incubating the bacterial strains with 0.125 mg/mL of the anti-BCAL2645 antibody for 1 h at room temperature prior the contact with the CFBE41o- cell monolayers. The results obtained indicate a remarkable decrease of invasion when the bacterial cells were treated with the antibody, with a reduction close to 85% in the number of WT bacteria found intracellularly in CFBE41o- epithelial cells. The reduction observed for the Δ*BCAL2645* strain was about 35% (Figure 5B).

## 4. Discussion

Previous work from our group led to the identification of the BCAL2645 protein among 16 surface exposed proteins of *B. cenocepacia* J2315 and was confirmed as being immunogenic against the sera of CF with a previous record of Bcc infection [23]. This protein belongs to the OmpA family of proteins, known for their importance in the pathogenesis of bacteria, such as bacterial adhesion and invasion, intracellular survival, biofilm formation and stimulation of proinflammatory cytokines [26,27,28,29,30]. OmpA proteins are targets of the host immune system and are regarded as good candidates for the development of immunotherapies against bacterial strains resistant to multiple antibiotics. In the present work, we aimed to functionally characterize the BCAL2645 protein and to investigate its potential use in immunoprotective therapies. With this purpose, a mutant with the *BCAL2645* gene deleted was constructed and a polyclonal goat antibody anti-BCAL2645 was produced. The mutation was performed by a double recombination strategy that resulted in the deletion of the encoding gene. The polyclonal goat antibody anti-BAL2645 was produced by a commercial company using an endotoxin-free recombinant 6×his-tagged BCAL2645 protein produced in our lab. The purified antibody was found to be highly specific against the BCAL2645 protein using total protein extracts from *B. cenocepacia* K56-2. The antibody was also used to confirm the absence of the BCAL2645 protein in the mutant strain. Despite their association with virulence, OmpA proteins also play diverse vital roles in bacteria, ranging from the integrity of the outer membrane to the passage of molecules through the membrane [61,62]. Our results show that no significant differences between the WT and mutant strain were found when growing in LB medium or M9 minimal medium, revealing that the mutant strain has no growth defect. Studies have found that this family of proteins are important in the maintenance of the outer membrane integrity, with its transmembrane domain acting as a virulence factor, and the periplasmic C-terminal domain being associated with the peptidoglycan hence helping to maintain outer membrane integrity [61,62]. In *E. coli*, mutants lacking an OmpA protein were more sensitive to SDS, acidic environments, high osmolarity and ethanol [53,63]. However, no differences were detected between *B. cenocepacia* K56-2 WT and the Δ*BCAL2645* mutant upon exposure to the presence of 0.03% (*w*/*v*) SDS, 426 mM NaCl, 3% (*v*/*v*) ethanol, or heat-shock at 50 °C (data not showed). In addition, there were no differences in the resistance profiles to chloramphenicol, trimethoprim and tetracycline, despite the association of porins and antibiotic resistance [53,64]. A total of 10 OmpA-like proteins are encoded in *B. cenocepacia* genomes; it is quite possible that the absence of one OmpA is compensated by other OmpA protein to maintain control of the efflux and influx from the cell, as well as the stability of the outer membrane and resistance to stressors. Colonies of the mutant and WT strain exhibited differences in morphology. After 48 h of growth on the surface of LB, solid medium colonies of the WT strain developed a characteristic wrinkled texture, while colonies of the mutant strain showed a smooth texture. The formation of smooth colony morphology and impairment of later stages of biofilm formation was previously attributed to the impairment of exopolysaccharide biosynthesis [65,66]. Whether the OmpA-like BCAL2645 protein is involved in exopolysaccharide biosynthesis is unknown. One of the most common roles of OmpA-like proteins is related to biofilm formation [28]. Biofilms, along with the intrinsic resistance to antibiotics, have been described as the pillars for Bcc chronic infections [67]. When growing in biofilms, bacteria tend to exhibit higher tolerance to antibiotics [11], some evolve to persister cells capable of surviving higher antibiotic concentrations [68] and the whole biofilm community is protected from professional cells of the immune system, like macrophages [69]. Nevertheless, recent studies suggest that Bcc tend to be present as single cells or small clusters in the mucus layers of the lungs or within phagocytes, instead of relying on biofilm-like structures [60,70]. Our results strongly suggest that this protein plays a role in the later stages of biofilm formation, as the mutant strain is unable to form a thick and mature biofilm. This alteration is likely linked to a decrease in adhesion, as it was previously observed that a decrease in biofilm was associated with a decrease in adhesion to human epithelial cells [49]. The luminal surface of the respiratory tract is covered by mucins that constitute the first anchor points for bacteria when infecting the host respiratory tract [56]. The *B. cenocepacia* Δ*BCAL2645* strain was impaired in adhesion to mucins, consistent with roles played by other OmpA proteins in adhesion [71]. The attachment to lung epithelial cells is a fundamental step in the infection process of Bcc, with some studies demonstrating selected proteins as playing important roles in this process [49,72]. Our results show that despite the decreased in vitro adhesion to mucins exhibited by the mutant strain, an increased adhesion to the CF human bronchial epithelial cell line CFBE41o- was observed for the mutant. These findings suggests that the lack of BCAL2645 probably allows other proteins to be present in higher quantities at the bacterial cell surface, increasing the adherence to the cell line. On the other hand, if BCAL2645 plays a role on exopolysaccharide biosynthesis, its absence would lead to reduced exopolysaccharide at the bacterial cell surface with the consequent higher exposure of adhesins and more efficient binding to the epithelial cells. Invasion of epithelial cells is another key step of Bcc infection. *B. cenocepacia* J2315 has been reported to be able to invade cultured human respiratory epithelial cells [73]. Results obtained in this work suggest that the BCAL2645 protein plays an important role in the invasion process of *B. cenocepacia* into CF human bronchial epithelial cells, as the mutant lacking the encoding gene exhibited a decreased ability to invade CFBE41o- epithelial cells.

In a transcriptomic profiling using 9 different growth conditions, Sass et al. showed that the *BCAL2645* mRNA was more abundant in cells harvested at the stationary phase of growth in LB medium, as well as when growth was carried out under microaerophilic conditions [53]. Using a quantitative western blot approach with the antibody anti-BCAL2645, we were able to confirm that the protein is indeed present in higher amounts in cells at the stationary phase growth. Our results also indicate that the BCAL2645 protein is more expressed in cells cultured in ASM medium and microaerophilic conditions, recognized environmental conditions found by bacteria when infecting CF patients [53,74].

Passive immunotherapies are frequently suggested for the treatment of antimicrobial resistant pathogens and for patients with impaired immune systems that are unable to produce an adequate immune response to active immunization. This is the case of the OprF from *Pseudomonas aeruginosa*, for which egg yolk-specific antibodies against the recombinant OprF were used for the immunization of burned mice. Previously immunized mice infected by *P. aeruginosa* exhibit survival rates of 87.5% compared to 25% for mice immunized with a control IgY [75]. In this work the polyclonal antibody raised against BCAL2645 was used to treat *B. cenocepacia* prior to in vitro adhesion to mucins and to epithelial cells, leading to reductions of 70% of *B. cenocepacia* adhering to mucins and a reduction of 75% in the number of *B. cenocepacia* adherent bacteria to epithelial cells. The treatment of bacterial cells with the antibody led to a remarkable reduction of 85% in the total number of bacteria capable of invading epithelial cells. These results highlight the potential of this antibody on passive immunization therapies, which emerged as attractive approaches to treat bacterial infections [76].

## 5. Conclusions

The BCAL2645 of *Burkholderia cenocepacia* is here characterized as a multifunctional protein affecting the latter stages of biofilm formation, helping the adherence to mucins, and playing an important role in the invasion of lung epithelial cells. The protein is more expressed in the bacterial cell cultures at the latter stages of growth and even more abundant in conditions mimicking those found in the lungs of CF patients. The protein expression pattern was altered when the cells were grown in microaerophilic conditions with the protein being highly expressed from early stages. These results underline the importance of this protein as an important virulence factor with multiple roles in pathogenesis. The inhibitory effect of an anti-BCAL2645 antibody was also tested, decreasing the number of adhered and invading bacteria by more than 70%, indicating its potential for the development and use of anti-BCAL2645 antibodies for passive immunization therapies to prevent infections by bacteria of the Bcc. Future studies using in vivo models will be pursued to further revaluate BCAL2645 as a protective immunotherapy against Bcc infections.

## Figures and Tables

**Table 1:** Bacterial strains and plasmids used in this work.

Strain or Plasmid	Genotype or Description	References or Source
**Strains**		
*E. coli* DH5α	F− Φ80*lac*Z∆M15 ∆(*lac*ZYA-*arg*F) U169 *rec*A1 *end*A1 *hsd*R17(r_k_−, m_k_+) *pho*A *sup*E44 *thi*-1*gyr*A96 *rel*A1 λ−	Invitrogen
*E. coli* BL21 (DE3)	F− *omp*T *hsd*SB (rB−mB−) *dcm gal* λ(DE3)	Stratagene
*B. cenocepacia* K56-2	Cystic fibrosis clinical isolate (Toronto, Canada); ET12 lineage	[36]
*B. cenocepacia* K56-2 ΔBCAL2645	*B. cenocepacia* K56-2 with the *BCAL2645* gene deleted	This study
**Plasmids**		
pSAS36	pET23a+ containing the *BCAL2645* gene, cloned downstream of the T7 promoter and upstream of the C-terminal 6× His-Tag, Ap^r^	[23]
pIN290	Suicide plasmid derived from pIN11 [37], *sacR/B* gene, RK2 *ori*T, I-SceI and encoding a DsRed fluorescent protein; Cm^r^	Kindly provided by Dr. Annette Vergunst
pDAI-SceI-SacB	pDA17 carrying the I-SceI gene and the counterselectable marker SacB, Tet^r^	[38]
pMLS7	pBBR1 ori, pS7 promoter, mob^+^, Tp^r^	[39]
pRK2013	Mobilizing vector, ColE1 tra (RK2)^+^, Km^r^	[40]
pAMS6	pIN290 with *BCAL2645* gene upstream and downstream regions cloned	This study
pAMS7	pMLS7 with *BCAL2645* gene cloned	This study

**Table 2:** List of primers used in this work, showing primers names, sequence, amplicon sizes and annealing temperatures. Restriction sites are represented as underlined sequences.

Primer Name	Primer Sequence (5′→3′)	Restriction Site	Product Size	Annealing T (°C)
UP-Nested BCAL2645	TAGATGTCGGCGTCGAGAATGC	-	2200 bp	60
LW-Nested BCAL2645	GAACATTTCCTTGACCGGATCGT	-		
UP-Upstream BCAL2645	ACCTCTAGACGGCAACAGCTTCAC	XbaI	640 bp	59.5
LW-Upstream BCAL2645	ACCGGATCCCTTGGATTCCTCTCTT	BamHI		
UP-Downstream BCAL2645	AAAGGATCCGCGCGACCACGAGA	BamHI	710 bp	59.5
LW-Downstream BCAL2645	ACCGAGCTCCGGTCGAGTAGAAG	SacI		
UP-BCAL2645Comp	CCCGGATCCATGAACATGAAAATCGC	BamHI	666 bp	64
LW-BCAL2645Comp	CCCAAGCTTTTACTGATGCTGTTGC	HindIII		

Abbreviations: UP, forward; LW, reverse; bp, base-pairs.

## Data Availability

Not applicable.
